# Hot Uniaxial Pressing and Pressureless Sintering of AlCrCuFeMnNi Complex Concentrated Alloy—A Comparative Study

**DOI:** 10.3390/ma17225457

**Published:** 2024-11-08

**Authors:** Tiago Silva, Pedro Simões, Augusto Lopes

**Affiliations:** 1 Department of Materials and Ceramic Engineering, CICECO-Aveiro Institute of Materials, University of Aveiro, 3810-193 Aveiro, Portugal; augusto@ua.pt; 2 Termolab—High Temperature Technology, 3750-309 Águeda, Portugal; pedro.simoes@termolab.pt

**Keywords:** complex concentrated alloys, microstructure, powder metallurgy, hot uniaxial pressing sintering, pressureless sintering, sintering atmosphere

## Abstract

External pressure is often applied during sintering to obtain materials with improved properties. For complex concentrated alloys (CCAs), this processing step is commonly performed in vacuum. However, this can promote the evaporation of elements and increase the oxide content, thereby degrading the properties of the alloy. In this study, we compared the microstructures and properties of AlCrCuFeMnNi CCA samples obtained by hot uniaxial pressing sintering (HPS) and pressureless sintering (PLS) using a helium atmosphere purified by an oxygen getter system. The powders were prepared from mixtures of CrFeMn, AlNi and Cu and sintered by HPS at 900 °C for 1 h with an applied pressure of 30 MPa and by PLS at 1050 °C for 1 h. The samples were characterised using X-ray diffraction, scanning and transmission electron microscopy, energy-dispersive X-ray spectroscopy, electron backscattering diffraction, density measurements and hardness tests. It was found that the oxygen getter system promoted oxygen partial pressure values at sintering temperatures similar to those of a mixture of 90% helium and 10% hydrogen. The HPS allowed us to obtain almost fully dense samples with a smaller average grain size and finer distribution of aluminium oxides than PLS. These differences increased the hardness of the samples sintered under pressure.

## 1. Introduction

Most metallic materials currently available for specific and general applications are based on a single principal element with small amounts of other elements to promote solid solution or precipitation strengthening. In the last decade, an increasing number of studies have been conducted on metallic materials with quasi-equimolar proportions of three or more elements [1,2,3]. Unlike conventional alloys, these alloys contain several major elements [1,2,3]. These materials, named complex concentrated alloys (CCAs), introduce new possibilities for developing materials with improved properties, such as higher hardness and strength at room and elevated temperatures, lower thermal conductivity, enhanced wear behaviour and improved corrosion resistance, for applications such as structural materials, coatings, tools and high-temperature components [1,2,3].

CCAs are defined as alloys containing three or more elements in concentrations between 5% and 35% [1,2,3,4] without a single dominant element. This definition includes alloys with a single phase (high entropy alloys) or different phases, such as ordered and disordered solid solutions (SSs), intermetallics (IMs), or a mixture of both [2]. The SS obtained in these alloys is usually classified by its atomic packing sequence as face-centred cubic (FCC), body-centred cubic (BCC), or hexagonal compact (HCP) [2].

Most CCAs reported in the literature are composed of Al, Co, Cr, Cu, Fe, Ni, Ti, V, Mn and, in some cases, refractory elements such as W and Mo. The studies show that Cu, Ni, Mn and Co elements favour the formation of FCC phases, while Al, Cr, W, V and Ti enhance the formation of BCC phases [2]. HCP phases are relatively uncommon and are usually based on rare earth elements (Sc, Y and lanthanides) [5].

Studies performed on AlCrCuFeMnNi CCA show that, as cast, this alloy can reach a hardness of approximately 500 HV, has good resistance to annealing softening and good oxidation resistance at room and high temperatures [6,7]. Arc melting is the principal processing method for producing CCAs based on Al, Cr, Cu, Fe, Mn and Ni [6,8,9,10,11]; however, powder metallurgy has also been used [12,13,14]. The latter has some advantages when compared to melting processing, such as the good homogeneity of powders; can be used in systems containing elements with a broad range of melting points; and can be easily adapted for mass production at a low cost [12,13].

Powder metallurgy is a solid-state process that usually includes mechanical alloying (MA) or milling, followed by shaping and a sintering cycle for the consolidation of the powder. Previous work on AlCrCuFeMnNi CCA obtained by pressureless sintering (PLS) showed that the formation of a liquid phase during sintering favours the coalescence and heterogeneous distribution of oxide particles, which decreases the hardness of the obtained samples [14]. However, the absence of a liquid phase during this thermal cycle by decreasing the temperature can cause poor densification, limiting the properties of CCA processed by PLS. Samples with high densification can be obtained by sintering without the presence of a liquid phase through techniques such as spark plasma or hot pressing sintering.

Spark plasma sintering (SPS) is one of the most common techniques reported in the literature for sintering CCAs obtained by MA [1,15]. Despite the very fast sintering cycles and heating rates, this technique requires highly specialised equipment [16,17]. Hot pressing sintering (HPS) uses relatively simple equipment compared to SPS and can produce samples with high relative densities and good mechanical properties. The use of external pressure allows the use of lower sintering temperatures, avoiding the formation of a liquid phase and the segregation of alloying elements [18,19]. Most of the studies on CCAs reported in the literature were performed under low vacuum [19,20] or argon [19] sintering atmospheres and applied uniaxial pressures of approximately 10 to 80 MPa [21,22]. However, these atmospheres often give rise to the formation of oxides [19]. Al-, Mn- and Cr-containing alloys are usually more sensitive to oxidation during sintering [23], requiring lower oxygen partial pressure atmospheres such as high vacuum, dry nitrogen, argon, helium and hydrogen. However, high-vacuum atmospheres can cause the evaporation of elements such as Mn, Al and Cu [24]. An argon atmosphere is by far the most used choice for the PLS of CCAs [25,26,27]. Hydrogen-containing atmospheres are often used for sintering steels and Ti alloys [28,29]. Nitrogen atmospheres are commonly used for sintering aluminium alloys [30,31]. As an alternative, some studies used a getter material (sacrificial metal, such as Al, Ti, Zr, Mg and Ca), which reacts with residual oxygen during sintering, decreasing the oxidation of the alloy during this processing step [32,33,34,35]. The getter system is usually placed in contact with the atmosphere to be purified (external getter) or integrated into the composition of the material to be processed (internal getter). For example, Wolff et al. [32] explored the use of Mg as an external getter and the addition of Ca to the alloy to be processed as an internal getter. They found significant positive effects during sintering, such as an increased relative density and improved mechanical properties.

The main objective of this study is to compare the microstructure and properties of AlCrCuFeMnNi CCA samples obtained by HPS and PLS using an oxygen getter system during the sintering process.

## 2. Materials and Methods

CCA powder with a nominal composition of AlCrCuFeMnNi was produced by wet milling mixtures of AlNi, CrFeMn and Cu powders and used to prepare samples by hot uniaxial pressing and by uniaxial pressing, followed by pressureless sintering. AlNi powder with a purity higher than 99.5% and an average particle size of approximately 60 µm was supplied by Alfa Aesar (Ward Hill, MA, USA) The CrFeMn precursor powders were supplied by Alfa Aesar, with a purity higher than 99.5% and a particle size lower than 45 µm. This alloy was produced by melting Cr, Fe and Mn in a 90% argon and 10% hydrogen atmosphere at 1350 °C for 30 min. After melting, the slag was removed and the material was crushed to a size lower than 350 µm. The Cu powder was heat treated at 1000 °C for 1 h under the same atmosphere. These treatments were performed to reduce the oxygen content of the initial material. The powders were weighed in equiatomic proportions and wet-milled in toluene for 15 h in an atmosphere of 90% helium and 10% hydrogen using planetary milling equipment (Rethch PM100, Haan, Germany) operating at 250 rpm with a ball-to-powder ratio of 10:1. Steel vials and balls were used as milling media. After sieving to a 75 µm mesh, the obtained powder was used to prepare samples with a height of 4 mm and diameter of 10 mm by applying a uniaxial pressure of 759 MPa, followed by pressureless sintering at 1050 °C for 60 min (PLS samples).

**Figure 1:**
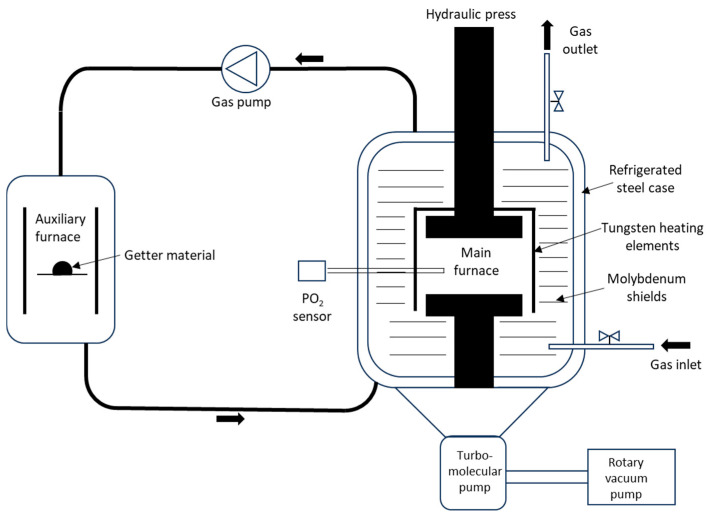
A schematic representation of the system used for sintering.

The powder was also used to obtain samples with a diameter of approximately 20 mm and a height of 3 mm by hot uniaxial pressing using a graphite mould, a constant load of 30 MPa and a maximum temperature of 900 °C for 1 h (HP samples). Both sintering processes were performed in a purified helium atmosphere with an oxygen getter system installed in an auxiliary furnace connected to the main chamber containing the samples (Termolab, Águeda, Portugal), as shown in Figure 1. Al powder was used as a getter because of its high tendency to form stable oxides at low oxygen partial pressures, low cost and low vapour pressure at the sintering temperatures used in this study [24].

After a vacuum cycle until around 10^−9^ atm, helium gas (supplied by Airliquid, Madrid, Spain, with a purity higher than 99.999%) was introduced into the main furnace and forced by a pump to circulate in a closed-circuit through the getter material. During the sintering of the samples, the temperature of the auxiliary furnace and the gas pressure in the system were kept constant at, respectively, 700 °C and 1 atm. The partial pressure of oxygen (*PO*_2_) inside the main chamber was measured using a zirconium oxygen sensor (Econox-CarboProbe DS, Alle, Switzerland). The electromotive force generated by this sensor due to the difference in the oxygen concentrations in the atmospheres inside and outside the furnace was measured with a digital multimeter and used to calculate the *PO*_2_ values trough the Nerts equation as follows [36]:

(1) $${PO}_{2}=0.209e^{-46.21E/T}$$

where *E* is the electromotive force of the sensor in mV, and *T* is the absolute temperature.

For comparison purposes, the *PO*_2_ values were also measured without the getter system for atmospheres of high vacuum (around 10^−9^ atm, using a turbomolecular pump (Leybold Turbovac 350i, Cologne, Germany) and a continuous flow of argon, helium and a mixture of 90% helium and 10% hydrogen (all with a purity higher than 99.999% and supplied by Airliquid, Madrid, Spain).

The powder and sintered samples were characterised by X-ray diffraction (XRD) using a PANalytical X’pert Pro diffractometer operating with Cu-Kα radiation, 2θ from 10 to 100°, a step size of 0.026° 2θ and a scan stem size of 597 s (Malvern, UK), scanning electron microscopy (SEM, Hitachi SU-70, Tokyo, Japan) and energy-dispersive X-ray spectrometry (EDS, Bruker Quantax, Billerica, MA, USA and Oxford Ultim Max systems, Oxfordshire, UK). The particle size distributions of the powder mixture after milling were analysed using laser scattering (Horiba LA-960, Kyoto, Japan). The sintered samples were also characterised by electron backscattering diffraction (EBSD, Bruker Quantax Cryst Align, Billerica, MA, USA), transmission electron microscopy (TEM, JEOL 2200FS, Tokyo, Japan), density measurements and indentation tests. For SEM, EDS and EBSD analyses, the samples were polished to a mirror finishing using 0.06 µm of colloidal alumina. For TEM analysis, the samples were first mechanically ground to a thickness of 20 µm and ion-beam-polished until a hole was formed (Gatan 691 Precision ion polishing system, Pleasanton, CA, USA) using 1.5 kV to 4 kV and angles between 4° and 2°.

The densities of the samples were calculated by measuring the weight and dimensions (samples before sintering) and using the Archimedes principle with water (samples after sintering). The mechanical properties of the sintered samples were evaluated using Vickers’ hardness tests (2 kgf load, Wilson VH1102, Lake Bluff, IL, USA) and nanoindentation tests (CSM-MST, Graz, Austria) with a Berkovich pyramid-shaped indenter tip. Nanoindentations were performed with a maximum load of 100 mN, a loading rate of 20 mN/min, a dwell time of 10 s and an unloading rate of 200 mN/min. The nanohardness (*H*) and Young modulus (*E*) values were calculated from the unloading curves using Oliver–Pharr analysis [37]. The H value was calculated as the ratio between the maximum load (*F_m_*) and projected area of the indenter impression (*A_p_*):

(2) $$H=\frac{F_{m}}{A_{P}}$$

For the Berkovich indenter

(3) $$A_{p}=f\left({h_{c}}^{2}\right)=24.5{h_{c}}^{2}$$

where $h_{c}$ is the contact depth at the maximum load, which can be calculated as follows:

(4) $$h_{c}=h_{max}-\varepsilon \frac{F_{m}}{S}$$

where *h_max_* is the displacement at the maximum load, *ε* is a constant related to the indenter geometry [38] and *S* is the unloading stiffness at the peak load, which can be calculated from the slope of the load–displacement curve using the following equation:

(5) $$S=\frac{dF}{dh}=\beta \frac{2}{\sqrt{\pi }}E_{r}\sqrt{A_{p}}$$

where *β* is equal to 1.034 for a Berkovich indenter [38], and *E_r_* is the reduced Young modulus given by

(6) $$\frac{1}{E_{r}}=\frac{1-\nu^{2}}{E}+\frac{1-{\nu_{i}}^{2}}{E_{i}}$$

*E* and *E_i_* are the Young moduli of the specimen and indenter, respectively, while *ν* and *ν_i_* are the Poisson ratios of the specimen (assumed as 0.3) and the indenter [38].

## 3. Results and Discussion

### 3.1. Powder Characterisation

**Figure 2:**
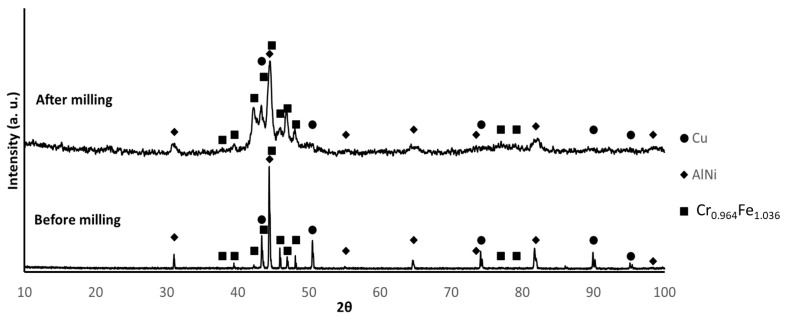
XRD patterns of the powder before and after milling (PDF card index: Cu: (01-070-3039); AlNi (04-012-6340); Cr_0.964_Fe_1.036_ (01-071-7533) [39]).

The X-ray diffractograms of the powder before and after milling are shown in Figure 2. The position and intensity of the diffraction maxima were as expected for the mixture of Cu, AlNi and CrFeMn. Because the diffraction file (PDF) data for CrFeMn were not available, the corresponding peaks were identified based on the experimental diffractograms of the alloy prepared by melting, which was almost coincident with the diffraction pattern tabulated for the Cr_0.964_ Fe_1.036_ phase. The observed broadening of the diffraction maxima after milling was a result of the higher lattice strain and smaller crystallite size promoted by the milling process.

**Figure 3:**
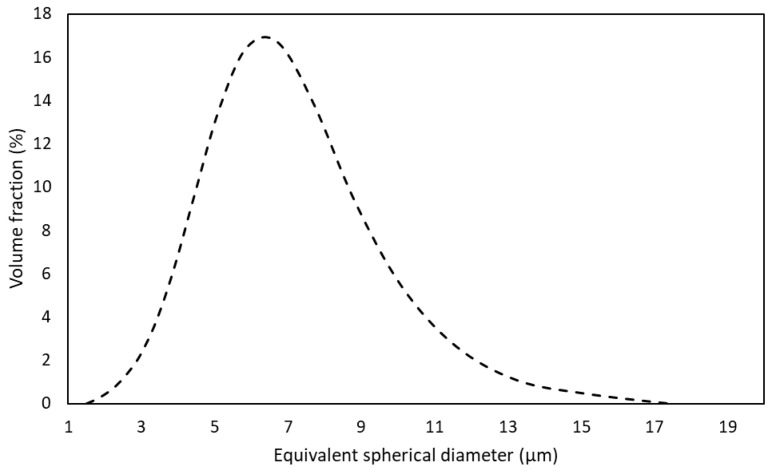
The particle size distribution of the powder after milling.

**Figure 4:**
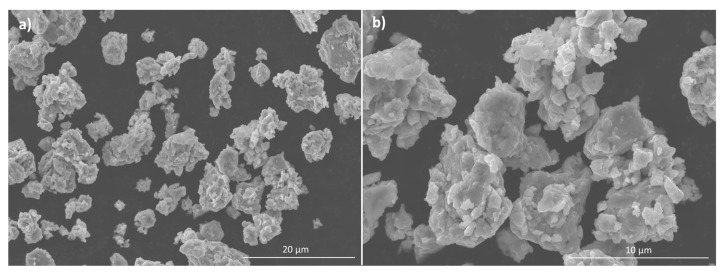
(**a**,**b**) SEM images at different magnifications of the powder after milling.

Laser scattering analysis of the powder after milling (Figure 3) showed a unimodal size distribution, with a maximum at around 7 µm and values lower than about 17 µm. The powder consisted mainly of agglomerates of particles with sizes less than around 3 µm, high chemical homogeneity and an equiaxed shape (Figure 4). These results are identical to those reported in [14] for the AlCrCuFeMnNi powders prepared under similar conditions.

### 3.2. Characterisation of the Sintered Samples

**Figure 5:**
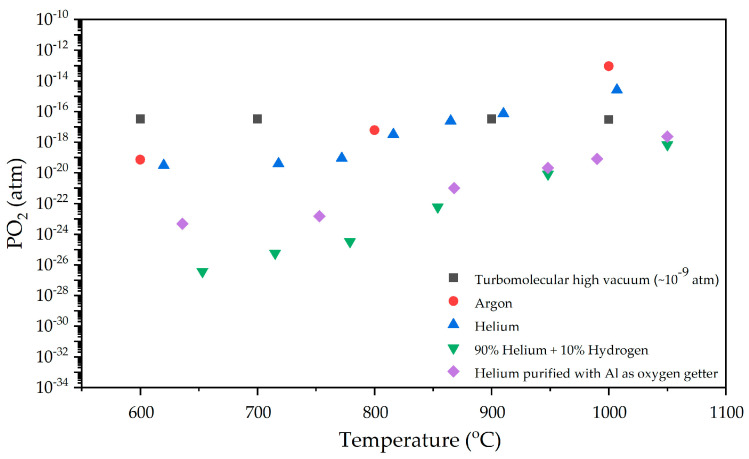
The oxygen partial pressure measured at different temperatures for the tested atmospheres.

Figure 5 shows the measured oxygen partial pressures at different temperatures for the studied atmospheres. The results show that the *PO*_2_ values for turbomolecular high vacuum are close to those obtained for argon and helium atmospheres. It is also possible to verify that the mixture of 90% helium with 10% hydrogen presents the lowest *PO*_2_ values at all tested temperatures, showing the high reducing power of hydrogen as a result of its high reactivity with oxygen.

The decrease in the *PO*_2_ values measured for helium purified with the oxygen getter system compared with the values exhibited by the helium atmosphere without purification shows that the use of aluminium as a getter material is quite efficient, allowing for a decrease in the oxygen concentrations in the sintering atmosphere close to those achieved with a mixture of 90% helium and 10% hydrogen, particularly at the temperatures used in this work for sintering.

**Figure 6:**
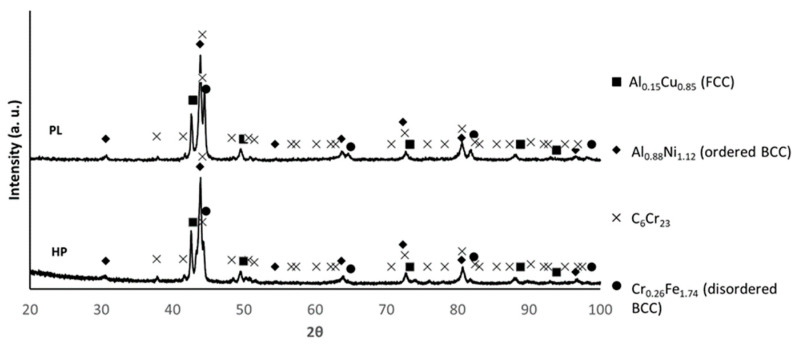
XRD patterns of the PLS and HP samples (PDF card index: Al_0.15_Cu_0.85_: (01-077-6740); Al_0.88_Ni_1.12_ (04-002-1233); C_6_Cr_23_ (04-004-3124); Cr_0.26_Fe_1.74_ (00-034-0396) [39]).

Figure 6 shows the XRD diffractograms of the samples after hot pressing and pressureless sintering. The patterns are similar, allowing us to identify in both samples the presence of C_6_Cr_23_, Al_0.88_Ni_1.12_ (ordered BCC), Cr_0.26_Fe_1.74_ (disordered BCC) and Al_0.15_Cu_0.85_ (FCC) [6,7,11].

**Figure 7:**
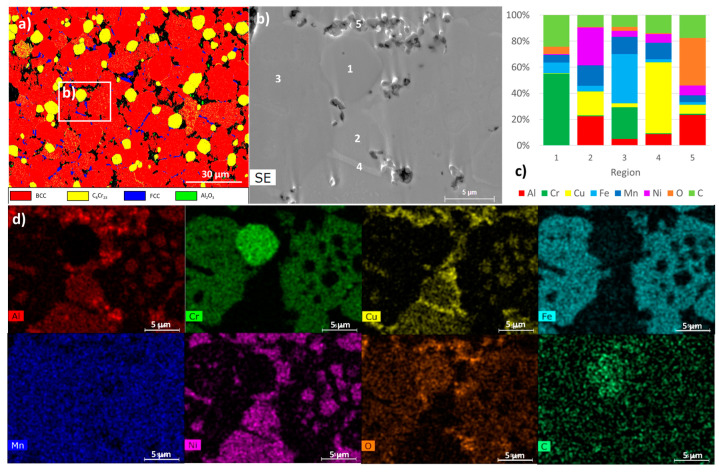
The results obtained for the PLS sample: (**a**) EBSD phase map of BCC, C_6_Cr_23_, FCC and Al_2_O_3_; (**b**) an SEM image of the area defined in (**a**); (**c**) the results of quantitative analyses by EDS at the points (1, 2, 3, 4 and 5) identified in the SEM image; (**d**) the distribution maps (images below) of Al, Cr, Cu, Fe, Mn, Ni, O and C.

**Figure 8:**
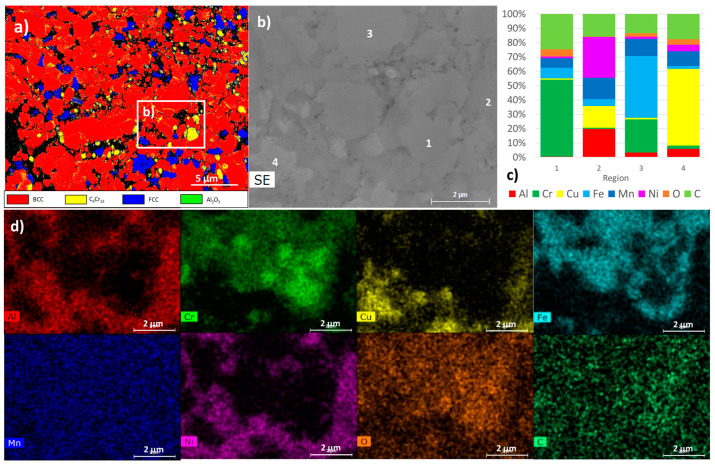
The results obtained for the HP sample: (**a**) EBSD phase map of BCC, C_6_Cr_23_, FCC and Al_2_O_3_; (**b**) an SEM image of the area defined in (**a**); (**c**) the results of quantitative analyses by EDS at the points (1, 2, 3 and 4) identified in the SEM image; (**d**) the distribution maps (images below) of Al, Cr, Cu, Fe, Mn, Ni, O and C.

The presence in both samples of C_6_Cr_23_, FCC and BCC phases was also detected by EBSD (Figure 7 and Figure 8). It should be noted that the BCC ordered and disordered phases present similar diffraction patterns, making it impossible to distinguish them using EBSD. However, the presence of both BCC phases was confirmed by the presence of areas rich in Al and Ni (ordered BCC phase) and areas rich in Cr and Fe (disordered BCC phase), as detected by EDS (points 2 and 3 in Figure 7 and Figure 8).

The presence of the carbide C_6_Cr_23_ phase was also confirmed by EDS (point 1 in Figure 7 and Figure 8). This phase has high hardness, but its presence reduces the amount of Cr in the solid solution and can decrease the corrosion resistance of the alloy [40]. The carbon present can be originated from the contamination of the initial powders, milling process and, possibly, the graphite parts of the furnace used for sintering.

The FCC Cu-rich phase (point 4 in Figure 7 and Figure 8) is present in lower amounts than the BCC phases. Comparing the two samples, it is observed that this phase has a different distribution. In the sample, PLS (Figure 7) occurs essentially at the BCC grain boundary and presents an elongated shape and size smaller than the other phases. In the HP sample (Figure 8), this phase occurs with a more rounded shape and size closer to those of the BCC and carbide phases. Another important difference between the samples is the grain size. In fact, the HP sample presents a finer microstructure, with grain sizes around one order of magnitude smaller than those in the PLS sample (average sizes of 1.2 µm and 10.5 µm, respectively).

**Figure 9:**
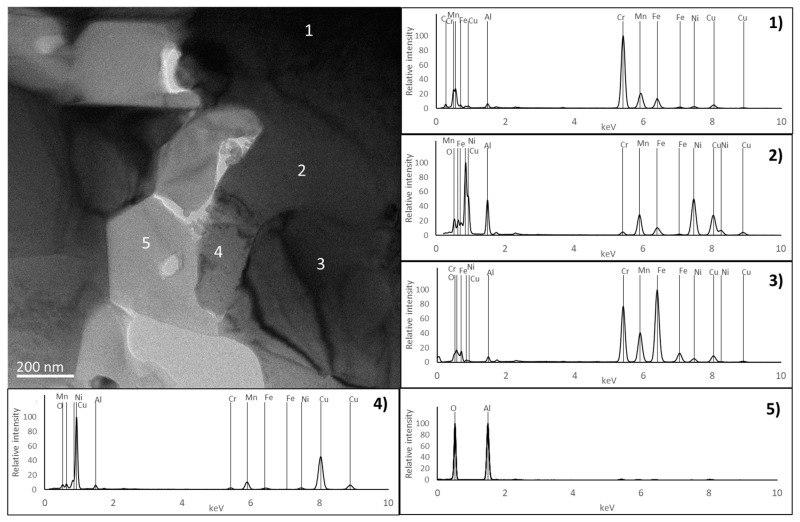
A TEM image of the PL sample and EDS spectra from the regions identified with numbers 1, 2, 3, 4 and 5.

**Figure 10:**
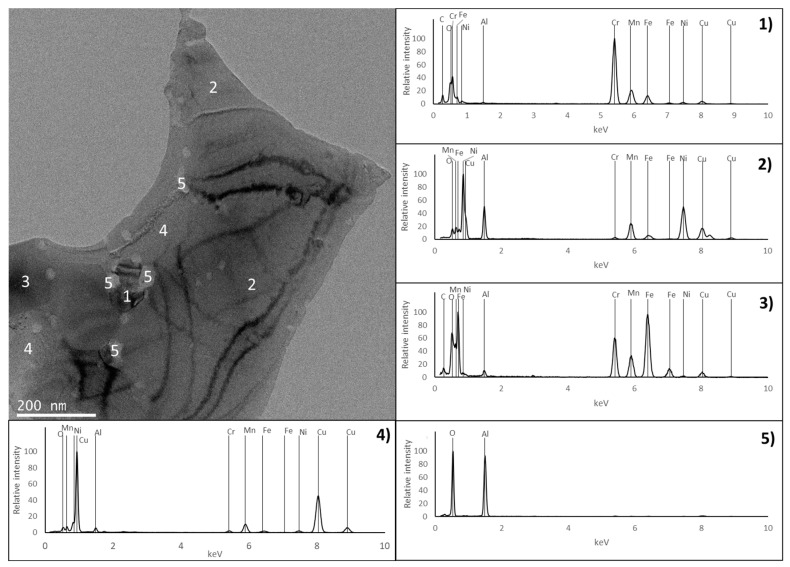
TEM image of the HP sample and EDS spectra from the regions identified with numbers 1, 2, 3, 4 and 5.

The presence of an Al-rich oxide phase (point 5 in Figure 7) was also observed in the PLS sample in the form of particles that are usually located at the grain boundaries. This phase was not detected in the HP sample by EDS or EBSD. However, a more detailed analysis of the microstructure using TEM (Figure 9 and Figure 10) revealed the presence of aluminium oxide particles in both samples, but with very different sizes. In the PLS sample, these particles present a size of approximately 300 nm, and in the HP sample, they are around 20 nm. This reduced size justifies the non-detection of the oxide phase in the HP sample by EDS or EBSD.

The analysis by TEM also confirmed the presence of regions rich in Cr and C (corresponding to the C_6_Cr_23_ phase), rich in Al and Ni (corresponding to the ordered BCC phase), rich in Cr and Fe (corresponding to the disordered BCC phase) and rich in Cu (corresponding to the FCC phase), as detected by EBSD, SEM and XRD. In all these regions, the spectra acquired for the PLS and HP samples are very similar, indicating that the constituent phases are identical.

Table 1 shows the phases detected after sintering and the values of density, average grain size, Vickers hardness, nanohardness and Young’s modulus measured for the PLS and HP samples. The relative densities were calculated from the measured density and the theoretical density value used in a previous study [14]. For comparative purposes, we also present the values reported in the same work for PLS samples produced with identical processing conditions to those used in this study, but using a sintering atmosphere of 90% helium and 10% hydrogen. As can be seen, the density, constituent phases, grain size and Vickers hardness of the PLS samples sintered with each of these atmospheres are similar, showing that the use of the oxygen getter system can be an alternative to the sintering atmosphere with hydrogen. However, the getter system has the advantages of very low gas consumption due to the use of a closed-circuit system and reduces the risk of hydrogen embrittlement or water vapour entrapment phenomena when atmospheres with hydrogen are used [41].

**Figure 11:**
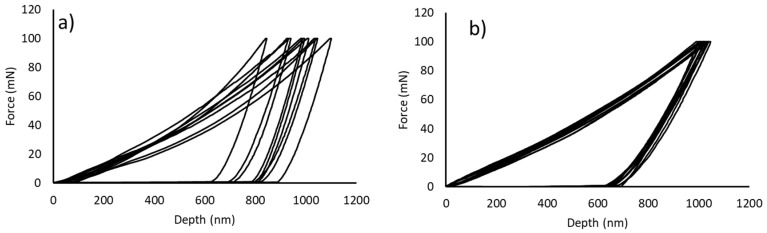
Nanoindentation load and unload curves of (**a**) a PLS sample and (**b**) an HP sample.

Another important conclusion is that the density of the HP samples is higher than that of the PLS samples and is close to 100%. The results also show that the Vickers hardness and nanohardness calculated from the loading and unloading curves (Figure 11) are higher for the HP samples than for the PLS samples. For both samples, the hardness values determined by nanoindentation are higher than those obtained by the Vickers tests, as reported in many studies. Nanohardness calculations consider a purely elastic contact to describe the interaction between the indenter and sample and use the projected area of contact at the maximum load instead of the residual indentation area [42]. The difference between these values increases for higher peak loads due to the larger plastic deformation of the sample in the contact area.

The higher hardness of the HP samples is justified by the higher relative density, smaller grain size and finer distribution of the oxide particles. In fact, the use of external pressure during sintering leads to a higher densification rate, as it increases the driving force for mass transfer and promotes changes in the particles shape and their arrangement through plastic deformation and creep [43,44]. Therefore, similar relative density values can be achieved with lower temperatures than those used in pressureless sintering, resulting in less pronounced grain growth and, therefore, materials with smaller grain sizes [43,44]. Furthermore, external pressure can promote the breakdown of oxide films on the surface of the particles, which hinders densification [45]. This effect and the use of a lower sintering temperature prevent the coalescence of oxides that decreases the hardness of the sintered samples [14]. Indeed, it was found that the coalescence of oxides in the studied CCA occurs with the formation of a liquid phase at approximately 950 °C [14]. This is in agreement with the presence of coalesced oxide particles in the PLS samples (whose sintering temperature was 1050 °C) but not in HP samples (whose sintering temperature was 900 °C).

Despite the lower porosity, the HP sample has a lower Young modulus than that of the PLS sample. This difference may be attributed to the smaller grain size [46] and differences in the relative distribution of the constituent phases, particularly the aluminium oxide particles and FCC phase. Another effect of the average grain size is the larger differences between several nanoindentation curves and the higher dispersion of hardness and Young’s modulus values obtained for the PLS samples.

## 4. Conclusions

In this work, it was possible to obtain AlCrCuFeMnNi CCA samples with high relative densities through pressureless and uniaxial hot pressing sintering using a mixture of Cu, AlNi and CrFeMn powders and a purified helium atmosphere with an oxygen getter system.

It was found that the use of the oxygen getter system led to partial oxygen pressures during sintering identical to those obtained with a mixture of 90% helium and 10% hydrogen, without significant changes in the phases, microstructure and properties of the obtained material.

After sintering, the HP and PLS samples exhibited the same phases but different microstructures. The HP samples showed a higher relative density, an average grain size approximately one order of magnitude lower and a finer distribution of aluminium oxides than the PLS samples. These differences resulted in higher Vickers’ hardness and nanohardness values for the HP samples.

The differences observed in the size and distribution of oxide particles were attributed to the combined effect of the applied external pressure and the lower sintering temperature in the HP samples, which prevented their coalescence, as observed in the PLS samples.

## Figures and Tables

**Table 1:** Values of density, grain size, detected phases, Vickers’ hardness, nanohardness and Young’s modulus for HP and PLS samples sintered with helium purified with the oxygen getter system and PLS sample sintered with an atmosphere of 90% He-10% H_2_ [14].

	HP	PL (Purified Helium)	PL (90%He + 10%H_2_) [14]
		Before sintering	
Density (g/cm^3^)	-	4.35 (60.1%)	4.40 (60.8%)
		After sintering	
Density (g/cm^3^)	6.87 (99.7%)	6.48 (95.9%)	6.50 (96.1%)
Phases	C_6_Cr_23_, Al_0.88_Ni_1.12_, Cr_0.26_Fe_1.74_, Al_0.15_Cu_0.85_ and Al_2_O_3_	C_6_Cr_23_, Al_0.88_Ni_1.12_, Cr_0.26_Fe_1.74_, Al_0.15_Cu_0.85_ and Al_2_O_3_	C_6_Cr_23_, Al_0.88_Ni_1.12_, Cr_0.26_Fe_1.74_, Al_0.15_Cu_0.85_ and Al_2_O_3_
Average grain size (µm)	1.2	10.5	10.7
Vickers’ hardness HV_2_ (GPa)	4.47 ± 0.03	3.64 ± 0.04	3.63
Nanohardness (GPa)	6.03 ± 0.16	5.17 ± 0.55	-
Young’s modulus (GPa)	78 ± 4	138 ± 11	-

## Data Availability

The raw data supporting the conclusions of this article will be made available by the authors on request.

## References

[1] Gao M.C., Yeh J.W., Liaw P.K., Zhang Y. (2016). High-Entropy Alloys Fundamentals and Applications.

[2] Miracle D.B., Senkov O.N. (2017). A critical review of high entropy alloys and related concepts. Acta Mater..

[3] Gorsse S., Miracle D.B., Senkov O.N. (2017). Mapping the world of complex concentrated alloys. Acta Mater..

[4] Gorsse S., Couzinié J., Miracle D.B. (2018). From high-entropy alloys to complex concentrated alloys. C. R. Phys..

[5] Qiao J.W., Bao M.L., Zhao Y.J., Yang H.J., Wu Y.C., Zhang Y., Hawk J.A., Gao M.C. (2018). Rare-earth high entropy alloys with hexagonal close-packed structure. J. Appl. Phys..

[6] Soare V., Mitrica D., Constantin I., Popescu G., Csaki I., Tarcolea M., Carcea I. (2015). The mechanical and corrosion behaviors of as-cast and re-melted AlCrCuFeMnNi multi-component high-entropy alloy. Metall. Mater. Trans..

[7] Nguyen T., Huang M., Li H., Hong L., Yang S. (2022). Effect of Al content on microstructure and mechanical properties of As-cast Al_x_FeMnNiCrCu_0.5_ high-entropy alloys. Mater. Sci. Eng. A.

[8] Rao Z., Wang X., Wang Q., Liu T., Chen X., Wang L., Hui X. (2017). Microstructure, mechanical properties, and oxidation behavior of AlxCr0.4CuFe0.4MnNi high entropy alloys. Adv. Eng. Mater..

[9] Otto F., Dlouhy A., Somsen C., Bei H., Eggeler G., George E.P. (2013). The influences of temperature and microstructure on the tensile properties of a CoCrFeMnNi high-entropy alloy. Acta Mater..

[10] Chen H.Y., Tsai C.W., Tung C.C., Yeh J.W., Shun T.T., Yang C.C., Chen S.K. (2006). Effect of the substitution of Co by Mn in Al-Cr-Cu-Fe-Co-Ni high-entropy alloys. Ann. Chim. Sci. Mater..

[11] He J.Y., Liu W.H., Wang H., Wu Y., Liu X.J., Nieh T.G., Lu Z.P. (2014). Effects of Al addition on structural evolution and tensile properties of the FeCoNiCrMn high-entropy alloy system. Acta Mater..

[12] Sharma V., Kumar S., Mallick A. (2023). Light weight MnTiAlNiFe high-entropy alloy (LWHEA) fabricated by powder metallurgy process: Mechanical, microstructure, and tribological properties. Mater. Today Proc..

[13] Kumar A., Singh A., Suhane A. (2022). Mechanically alloyed high entropy alloys: Existing challenges and opportunities. J. Mater. Res. Technol..

[14] Silva T., Lopes A. (2024). Microstructural characterization of AlCrCuFeMnNi complex concentrated alloy prepared by pressureless sintering. Materials.

[15] Murty B.S., Yeh J.W., Ranganathan S. (2014). High-Entropy Alloys.

[16] Sharma N., Alam S.N., Ray B.C. (2019). Fundamentals of Spark Plasma Sintering (SPS): An Ideal Processing Technique for Fabrication of Metal Matrix Nanocomposites.

[17] Suárez M., Fernández A., Menéndez J.L., Torrecillas R., Kessel H.U., Hennicke J., Kirchner R., Kessel T. (2013). Challenges and Opportunities for Spark Plasma Sintering: A Key Technology Form a New Generation of Materials.

[18] Ren B., Zhao R., Jiang A., Yu Y. (2022). Microstructure and oxidation behavior of CoCr_x_CuFeMnNi high-entropy alloys fabricated by vacuum hot-pressing sintering. Micron.

[19] Stasiak T., Sow M.A., Addad A., Touzin M., Béclin F., Cordier C. (2022). Processing and characterization of a mechanically alloyed and hot press sintered high entropy alloy from the Al-Cr-Fe-Mn-Mo family. Miner. Met. Mater. Soc..

[20] Jia J., Zhang K., Jiang S. (2014). Microstructure and mechanical properties of Ti–22Al–25Nb alloy fabricated by vacuum hot pressing sintering. Mater. Sci. Eng. A.

[21] Chang S., Wang C., Huang K. (2023). Influence of the vacuum hot pressing process on the microstructure, mechanical properties, and material characteristics of CrCoNiTa_3_ alloys. Vacuum.

[22] Cheng H., Liu X., Tang Q., Wang W., Yan X., Dai P. (2019). Microstructure and mechanical properties of FeCoCrNiMnAl_x_ high-entropy alloys prepared by mechanical alloying and hot-pressed sintering. J. Alloys Compd..

[23] Hasegawa M., Seetharaman S. (2014). Chapter 3.3—Ellingham Diagram. Treatise on Process Metallurgy.

[24] Safarian J., Engh T.A. (2013). Vacuum Evaporation of Pure Metals. Metall. Mater. Trans. A.

[25] Yuhu F., Yunpeng Z., Hongyan G., Huimin S., Li H. (2013). AlNiCrFe_x_Mo_0.2_CoCu high entropy alloys prepared by powder metallurgy. Rare Met. Mater. Eng..

[26] Qiu X. (2013). Microstructure and properties of AlCrFeNiCoCu high entropy alloy prepared by powder metallurgy. J. Alloys Compd..

[27] Mane R., Panigrahi B. (2018). Effect of alloying order on non-isothermal sintering kinetics of mechanically alloyed high entropy alloy powders. Mater. Lett..

[28] Várez A., Levenfeld B., Torralba J.M., Matula G., Dobrzanski L.A. (2004). Sintering in different atmospheres of T15 and M2 high speed steels produced by a modified metal injection moulding process. Mater. Sci. Eng..

[29] Fang Z., Sun P., Wang H. (2012). Hydrogen argon sintering of titanium to produce high density fine grain titanium alloys. Adv. Eng. Mater..

[30] Schaffer G.B., Hall B.J., Bonner S.J., Huo S.H., Sercombe T.B. (2006). The effect of the atmosphere and the role of pore filling on the sintering of aluminium. Acta Mater..

[31] Pieczonka T., Schubert T., Baunack S., Kieback B. (2008). Dimensional behaviour of aluminium sintered in different atmospheres. Mater. Sci. Eng..

[32] Wolff M., Thomas E., Michael D. (2010). Sintering of magnesium. Adv. Eng. Mater..

[33] Yong H.K., Ryosuke O.S., Hiroshi N., Hirobumi W., Katsutoshi O. (1997). Removal of oxygen and nitrogen from niobium by external gettering. J. Alloys Compd..

[34] Palka K., Pokrowiecki R., Krzywicka M. (2019). Titanium for Consumer Applications.

[35] Christian G., Raquel C., Herbert D. (2016). The role of oxygen transfer in sintering of low alloy steel powder compacts: A review of the ‘‘Internal Getter’’ Effect. JOM.

[36] Hans-Dieter W., Wolfgang G. (1991). Fundamentals and principles of potentiometric gas sensors based upon solid electrolytes. Sens. Actuators B Chem..

[37] Oliver W.C., Pharr G.M. (1992). An improved technique for determining hardness and elastic modulus using load and displacement sensing indentation experiments. Mater. Res. Soc..

[38] Attar H., Ehtemam-Haghighi S., Kent D., Okulov I.V., Wendrock H., Bönisch M., Volegov A.S., Calin M., Eckert J., Dargusch M.S. (2017). Nanoindentation and wear properties of Ti and Ti-TiB composite materials produced by selective laser melting. Mater. Sci. Eng. A.

[39] (2024). Powder Diffraction File (PDF) Database 2024.

[40] Hongxia W., Yong C., Dongdong S., Changfeng C. (2020). Effect of Cr/Mo carbides on corrosion behaviour of Fe_Mn_C twinning induced plasticity steel. Corros. Sci..

[41] Bose A., German R.M. (1988). Sintering atmosphere effects on tensile properties of heavy alloys. Metall. Trans. A.

[42] Broitman E. (2017). Indentation hardness measurements at macro-, micro-, and nanoscale: A critical overview. Tribol. Lett..

[43] German R.M. (2014). Sintering: From Empirical Observations to Scientific Principles.

[44] Kang S.L. (2005). Densification, Grain Growth, and Microstructure.

[45] Kim W., Griffith W.M., Froes F.H. (1985). Surface oxides in P/M aluminum alloys. J. Met..

[46] Preisler D., Stráský J., Janovská M., Becker H., Harcuba P., Janeček M. (2020). Achieving high strength and low Young’s modulus by controlling the betastabilizers content in Ti-Nb-Ta-Zr-O alloys. MATEC Web Conf..

